# Effect of 15-hydroxyprostaglandin dehydrogenase gene on the proliferation of gastric cancer cell murine forestomach carcinoma

**DOI:** 10.3892/etm.2013.1404

**Published:** 2013-11-12

**Authors:** LIHUA LI, FU YANG, XIAOJIE WANG, JING HU, LIBO YANG, CHUNMING TANG, YUNHUA WU, KUN MIAO, RUI LIU, TAO SHOU

**Affiliations:** 1 Department of Oncology, The First Hospital of Yunnan Province, Kunming, Yunnan 650032, P.R. China; 2 Department of Hepatobiliary Surgery, The First Affiliated Hospital of Kunming Medical University, Kunming, Yunnan 650031, P.R. China

**Keywords:** 15-hydroxyprostaglandin dehydrogenase, gastric, transfection, proliferation, inhibition

## Abstract

The aim of the present study was to construct the eukaryotic expression vector pcDNA3.1/15-PGDH. The vector was used to transfect mouse murine forestomach carcinoma (MFC) cancer cells and observe the effects of 15-hydroxyprostaglandin dehydrogenase (15-PGDH) on the proliferation of MFC. pcDNA3.1/15-PGDH was constructed using gene recombination technology and the vector was used to transfect MFC cells to build a stable transfected cell strain. The expression levels of 15-PGDH in the transfected cells were detected using reverse transcription polymerase chain reaction. Optical Density (OD) values were determined using an MTT assay and used to draw cell growth curves. The effects of 15-PGDH on the proliferation of MFC were observed using a clone formation experiment. Following successful transfection by 15-PGDH, the relative expression levels of 15-PGDH in the MFC/15-PGDH cells were significantly higher (1.06±0.08) (P<0.01) compared with the empty plasmid-transfected group (0.22±0.01) and the untransfected group (0.21±0.01). Following transfection by 15-PGDH, cell growth was markedly inhibited. The MTT results showed that on days 4, 6 and 8, the 15-PGDH-transfected group had a low OD on average, which was significantly different (P<0.05) from the empty plasmid-transfected group or the untransfected group. The 15-PGDH-transfected group had a plating efficiency of 18%, and compared with the untransfected group (63%) and the empty plasmid-transfected group (59%), clone formation was significantly inhibited (P<0.01). Results of the present study indicate that transfection by 15-PGDH may significantly inhibit the proliferation and clone formation of MFC cells.

## Introduction

15-hydroxyprostaglandin dehydrogenase (15-PGDH) is a major rate-limiting enzyme in the biodegradation of PG. 15-PGDH was first identified as a novel colorectal cancer gene. Previous studies have indicated that 15-PGDH is closely associated with the occurrence and development of numerous tumors *in vivo* and a reduction in its expression level promotes the occurrence, development, infiltration and metastasis of tumors, as well as the formation of tumor blood vessels (1,2). Gastric cancer is a commonly observed malignant tumor worldwide. Although its incidence rate has decreased in developed countries, its mortality rate has not markedly declined. Among all malignant tumors, its mortality rate ranks second in the world and first in China. Studies show that 15-PGDH expression levels decrease significantly in gastric cancer cell strains, while the restoration of its expression induces the apoptosis of gastric cancer cells and blocks the cell cycle (3). These results further indicate that 15-PGDH may be important in inhibiting the occurrence, development, infiltration and metastasis of gastric cancer, and may become a new target for treatment of gastric cancer.

The aim of the present study was to restore 15-PGDH expression in cultured gastric cancer cells *in vitro* using gene transfection technology and to observe the inhibitory effect of 15-PGDH on the proliferation of gastric cancer cells. These results are likely to provide a theoretical basis for further *in vivo* studies and new indications for developing therapeutic drugs targeting gastric cancer.

## Materials and methods

### Reagents, cell strains and plasmid

15-PGDH primers were synthesized by Sangon Biotech Co., Ltd. (Shanghai, China). pMD18-T vectors and T4 ligase were purchased from Takara Bio, Inc. (Dalian, China). pcDNA3.1 eukaryotic expression vectors and Lipofectamine 2000 were purchased from Invitrogen Life Technologies (Carlsbad, CA, USA). BALB/c female mice (weighing 17–20 g) were purchased from the Experimental Animal Center of Yunnan (Kunming, China). The study was approved by the ethics committee of The First Hospital of Yunnan Province, Kunming, China.

### Construction of recombinant plasmids

Total RNA was extracted from the gastric tissues of each BALB/c mouse using TRIzol reagent. The RT-PCR amplification mixture contained the following primers: 15-PGDH, 5′-AAGCTTCTGCACCATGCACGTGA-3′ (upstream) and 5′-GCGGATCCTTCAGCTATGGCTAAC-3′ (downstream). AAGCTT and GGATCC are recognition sequences of the restriction endonucleases, *Hin*dIII and *Bam*HI. PCR products were combined with the pMD-18T simple vector to construct the cloning vector pMD18-T/15-PGDH, the double-enzyme cleavage cloning vector pMD18-T/15-PGDH with two restrictive endonucleases *Hin*dIII and *Xba*I and the empty vector pcDNA3.1. T4 DNA ligase connected with the target gene fragments of 15-PGDH and the linear plasmid of pcDNA3.1. The connected product (pcDNA3.1/15-PGDH) transforms *Escherichia coli* DH5α. Following this, positive clones were screened and plasmids were extracted for enzyme cleavage identification and sequencing identification.

### Cell transfection and screening of stable transfected cell strains

During culture of the gastric cancer cell murine foregastric carcinoma (MFC), 24-mesh cell culture plates were used to inoculate 1 ml culture solution containing 0.6×10^5^ cells, using Lipofectamine 2000 as a transfection reagent. MFC cells were transfected separately with pcDNA 3.1 and pcDNA 3.1/15-PGDH for 4–6 h. Next, complete culture solutions were changed, and following subculture, G418 was added to screen the stable transfected cell strains, which were frozen until use.

### Drawing of cell growth curves

The MTT assay was used to cultivate 15-PGDH-transfected, empty vector-transfected and parent cells. The culture plates were collected on days 1–6. Subsequently, 20 μl MTT (5 mg/ml) and 100 μl dimethyl sulfoxide were added to each mesh for detection of absorbance at 490 nm using an ELISA. This absorbance is also called optical density, which reflects the cell count and may be used to construct cell growth curves.

### Detection of 15-PGDH expression by reverse transcription polymerase chain reaction (RT-PCR)

The 15-PGDH-transfected, empty vector-transfected and untransfected cells were separately cultured to extract total RNA and to synthesize cDNA through reverse transcription. 15-PGDH genes were amplified by PCR. The amplified products were identified and analyzed via agarose gel electrophoresis.

### Clone formation

The 15-PGDH-transfected, empty vector-transfected and untransfected cells were separately cultured in a 5% CO_2_ incubator at 37°C and saturated humidity for 10–14 days until cloning formation was observed. Subsequently, the culture solutions were discarded. The cells were fixed by methanol at room temperature for 10 min and stained by 0.4% crystal violet for 10 min, followed by 3 washings with sterile water. The cells were observed under a light microscope (Guiguang Company, Guilin, China) to determine cell number, and images were captured of the colonies in each dish. Each experiment was repeated three times. Plating efficiency (PE) was calculated as follows: PE = (average colony count/inoculation count) × 100.

### Statistical methods

All data were analyzed using SPSS 13.0 (SPSS, Inc., Chicago, IL, USA) The means were compared between two and between multiple using t-tests and Student Newman Keuls, respectively. P<0.05 and P<0.01 were considered to indicate an extremely significant difference.

## Results

### Identification of recombinant plasmids by RT-PCR

**Figure 1:**
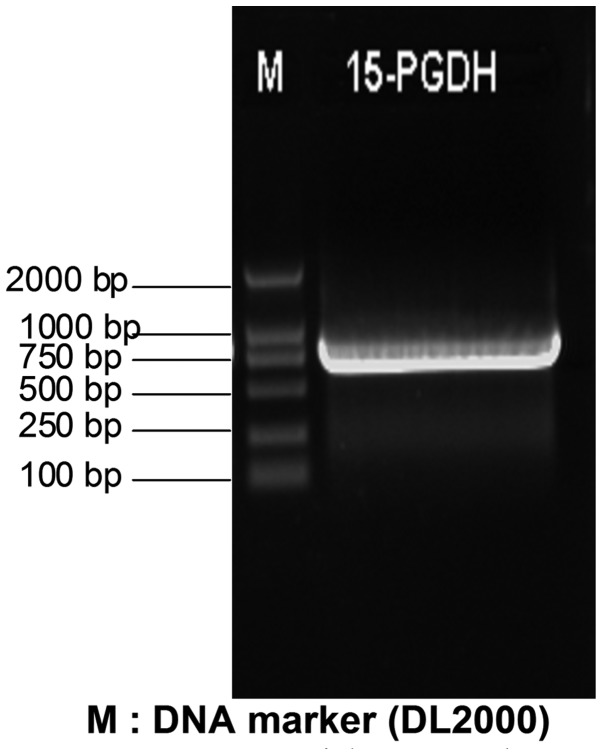
Identification of recombinant plasmid by polymerase chain reaction. 15-PGDH, 15-hydroxyprostaglandin dehydrogenase.

Competent DH5α was transformed by recombinant plasmids and 15-PGDH genes in colonies were amplified by PCR. The products were observed using 1% agarose gel electrophoresis. Observation of a specific band at ~845 bp represented the size of the target fragment (Fig. 1).

### Identification of the cloning vector pMD18-T/15-PGDH using enzyme cleavage

**Figure 2:**
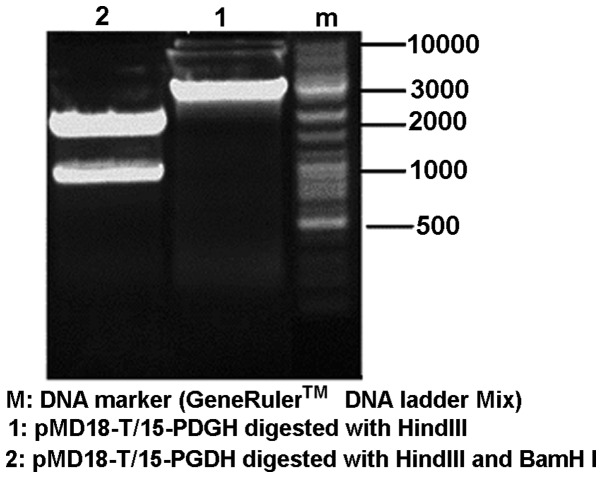
Identification of Pmd18-T/15-PGDH vector by restriction enzyme digestion. 15-PGDH, 15-hydroxyprostaglandin dehydrogenase.

After the pMD18-T/15-PGDH vector was cleaved by restriction endonucleases, *Hin*dIII and *Bam*HI, two DNA bands of ~2,700 and 845 bp in length were obtained (Fig. 2). This indicated that the gene sequence of 15-PGDH was successfully constructed into the *Eco*RV multiple clone site on the cloning vector pMD18-T.

### Identification of eukaryotic expression vector pcDNA3.1/15-PGDH using enzyme cleavage

**Figure 3:**
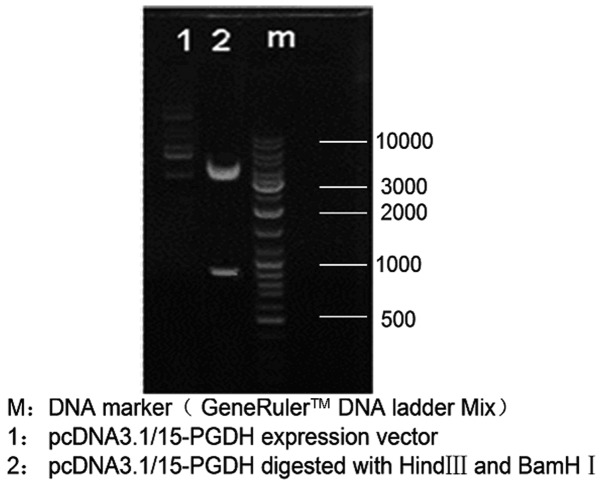
Identification of pcDNA3.1/15-PGDH vector by restriction enzyme digestion. 15-PGDH, 15-hydroxyprostaglandin dehydrogenase.

*Hin*dIII and *Bam*HI were used in double enzyme cleavage of the constructed vector pcDNA3.1/15-PGDH and the results (Fig. 3) indicated that the sequence of 15-PGDH was successfully constructed into the eukaryotic expression vector, pcDNA 3.1.

### Sequencing identification of recombinant plasmid

**Figure 4:**
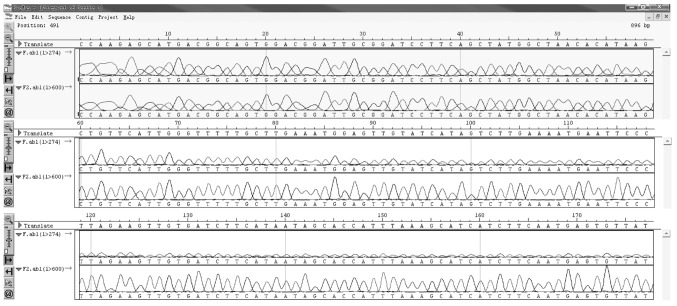
15-hydroxyprostaglandin dehydrogenase gene sequencing of reverse transcription polymerase chain reaction products.

The recombinant plasmid pcDNA3.1/15-PGDH was sequenced. The PCR-obtained 15-PGDH gene fragment was fully consistent with the sequence of the 15-PGDH open reading frame in the NCBI nr database, showing 100% consistency. A section of the sequence is showed in Fig. 4.

### Morphologic changes following transfection of MFC cells with the recombinant plasmids

**Figure 5:**
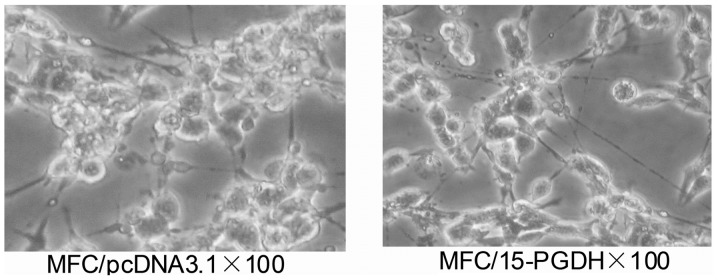
Morphous alteration of MFC (magnification, ×100). MFC, murineforestomach carcinoma; 15-PGDH, 15-hydroxyprostaglandin dehydrogenase.

Following transfection with 15-PGDH, significantly less cell stacking or multilayers were observed. The length of the cell body increased, cytoplasm transparency decreased and granular secretion increased (Fig. 5).

### Determination of cell growth curves

**Figure 6:**
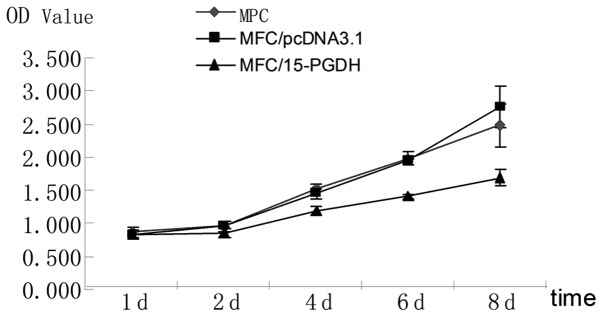
Cell proliferation curve. MFC, murine forestomach carcinoma; 15-PGDH, 15-hydroxyprostaglandin dehydrogenase.

The MTT results indicated that on days 4, 6 and 8, the growth of the 15-PGDH-transfected cells slowed down significantly (P<0.05) compared with the parent and empty vector-transfected cells (Table I). These results demonstrated that 15-PGDH exerts specific inhibitory effects on the growth of gastric cancer cells (Fig. 6).

### 15-PGDH expression following transfection of MFC cells with recombinant plasmids

**Figure 7:**
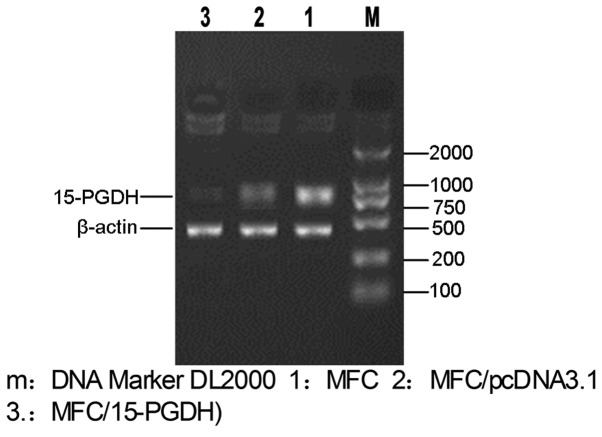
Changes in 15-PGDH gene expression following transfection. MFC, murine forestomach carcinoma; 15-PGDH, 15-hydroxyprostaglandin dehydrogenase.

MFC cells are a type of gastric cancer cells and have low expression levels of 15-PGDH. Following extraction of total RNA from cDNA of the empty plasmid-transfected and the recombinant plasmid-transfected cells (MFC/15-PGDH), the 15-PGDH genes were amplified. The results showed that 15-PGDH expression levels were low in MFC/pcDNA3.1 cells but high in MFC/15-PGDH cells with clear bands (Fig. 7).

### 15-PGDH transfection inhibits clone formation in MFC proliferation

**Figure 8:**
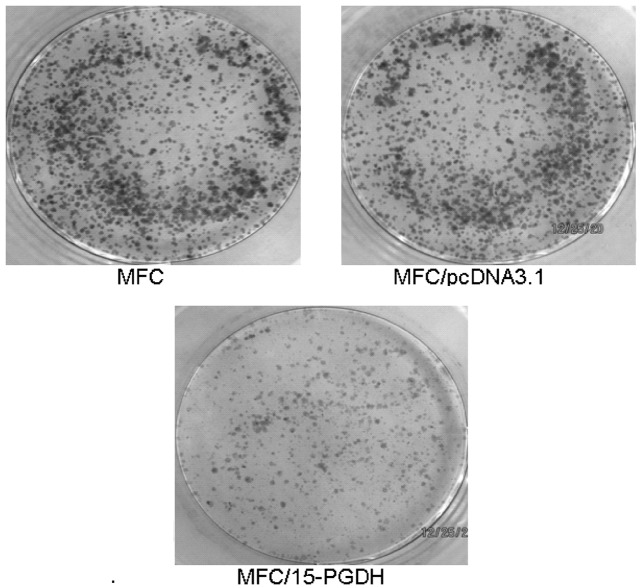
15-PGDH inhibits MFC proliferation by colony formation. MFC, murine forestomach carcinoma; 15-PGDH, 15-hydroxyprostaglandin dehydrogenase.

Cells in the control groups showed visible colonies after only 10 days of inoculation (Fig. 8). The experimental group (recombinant plasmid-transfected cells) had a PE of 18%, which is significantly lower (P<0.01) than the untransfected cells (63%) and the empty vector-transfected cells (59%). These results indicated that the expression of 15-PGDH may inhibit the proliferation of gastric cancer cells.

## Discussion

15-PGDH is a tumor suppressor gene, however, the mechanism by which it inhibits tumor proliferation is yet to be elucidated. Three theories have been put forward. Firstly, 15-PGDH inhibits tumor proliferation through antagonism of COX-2; COX-2 and the PGE2 it synthesizes may irritate the development of tumors by regulating their growth, proliferation and infiltration, the formation of blood vessels and the apoptosis of tumor cells (4). 15-PGDH may degrade PGE2 and thus exhibit natural antagonism against COX-2 (5–8). The second theory relates to the regulation of apoptosis. Previously, Li *et al*(3) reported that following transfection by 15-PGDH, the expression of proapoptosis genes, BAK, BAX and p53, increase, while the expression of anti-apoptosis genes, BCL-2 and BCL-XL, decrease. 15-PGDH may induce the apoptosis of SGC-7901 gastric cancer cells and inhibit the cell cycle. Thirdly, the irregular methylation in the promoter zone of 15-PGDH gene may cause its expression loss and after its methylation is reversed and 15-PGDH proteins may be reexpressed (9).

Expression of 15-PGDH is reduced, lost or its bioactivity is decreased in a number of malignant tumors (for example, colon cancer, gastric cancer, non-small cell lung cancer and prostate cancer). These may be the early events upon the occurrence of tumors (10,11). The occurrence and development of tumors may be inhibited if the expression of 15-PGDH is restored. This theory has been preliminarily proven in colon cancer and non-small cell lung cancer. Firstly, 15-PGDH genes were transferred into Vaco-400 colon cancer cells and injected into the forelimb of a nude mouse transplantation tumor experiment through injection into the forelimb. Although 15-PGDH expression was still lower than in normal cells, tumor growth slowed significantly compared with the control group (12). Secondly, in a A549 nude mice transplantation tumor model of non-small cell lung cancer, the restoration of 15-PGDH expression was also demonstrated to significantly inhibit the growth of tumors (13).

The existence of the correlation between 15-PGDH and gastric cancer is rarely reported. Specific studies have shown that 15-PGDH expression is reduced or lost in gastric cancer tissues and is significantly associated with the pathological type, degree of differentiation, the occurrence of distant metastasis and TNM staging (14,15). Whether restoration of 15-PGDH expression inhibits the growth and metastasis of gastric cancer cells has not been reported. To the best of our knowledge, the present study is the first discussion of this inhibition.

In the present study, a eukaryotic expression vector pcDNA3.1-PGDH was constructed. Subsequently, this recombinant plasmid was used to transfect gastric cancer MFC cells. The relative expression levels of 15-PGDH increased significantly compared with the empty vector-transfected group (control group). The effects of 15-PGDH transfection on the proliferation of MFC were observed. The growth curves show that on days 4,6 and 8, the growth of the 15-PGDH-transfected cells was markedly reduced, indicating that 15-PGDH has specific inhibitory effects on the growth of gastric cancer cells. Clone formation experiments revealed that PE was 18% in the recombinant plasmid-transfected cells and 63% in the untransfected cells, indicating that 15-PGDH expression may inhibit the proliferation of gastric cancer cells.

In general, the restoration of 15-PGDH expression inhibits the proliferation of gastric cancer cells. The reduction or loss of 15-PGDH expression is closely correlated with the occurrence of gastric cancer. Results of the present study indicate that 15-PGDH may play an important role in inhibiting the occurrence, development, infiltration and metastasis of gastric cancer cells. In addition, 15-PGDH may represent a novel target for the prevention and treatment of gastric cancer (16).

## Figures and Tables

**Table I:** MTT results of cells in each group (mean ± standard deviation).

Time (days)	MFC	MFC/pcDNA3.1	MFC/15-PGDH
1	0.865±0.062	0.822±0.067	0.832±0.064
2	0.953±0.066	0.975±0.091	0.846±0.074
4	1.534±0.082	1.452±0.062	1.173±0.072^a^
6	1.972±0.073	1.955±0.098	1.405 ±0.040^a^
8	2.477±0.319	2.755±0.324	1.689±0.116^a^

a P<0.05, MFC group vs. MFC/pcDNA3.1 group.

MFC, murine forestomach carcinoma; 15-PGDH, 15-hydroxyprostaglandin dehydrogenase.
